# Efficacy of vamorolone in treatment of Duchene muscle dystrophy. A meta-analysis

**DOI:** 10.3389/fneur.2023.1107474

**Published:** 2023-02-01

**Authors:** Rowan H. Elhalag, Karam R. Motawea, Nesreen Elsayed Talat, Samah S. Rouzan, Jaffer Shah

**Affiliations:** ^1^Faculty of Medicine, Alexandria University, Alexandria, Egypt; ^2^New York State Department of Health, New York, NY, United States

**Keywords:** Duchene muscular dystrophy (DMD), vamorolone, neuromuscular, glucocorticoids, steroid naïve

## Abstract

**Background and aim:**

Recent studies evaluated the role of vamorolone in treating Duchenne muscular dystrophy (DMD), so we aimed in our Meta-analysis to assess the efficacy of vamorolone in comparison with placebo and corticosteroids for treating DMD patients.

**Methods:**

We searched PubMed, Web of Science, Scopus, and Cochrane library databases. We included any randomized control trials and controlled observational studies that investigated the role of vamorolone in treating DMD patients. We used RevMan software, version 5.4. to perform our meta-analysis.

**Results:**

After a search of the literature, 4 studies were included in the meta-analysis; the total number of patients included in the study is 277 patients, 125 patients in the vamorolone group, 106 in the glucocorticoids group, and 46 in placebo (steroid naïve) group. The pooled analysis showed a statistically significant association between the vamorolone group and increased TTSTAND velocity, TTRW velocity and TTCLIMB velocity compared with the placebo group (MD = 0.04, 95% CI = 0.02–0.07, *p* = 0.002), (MD = 0.24, 95% CI = 0.11–0.37, *p* = 0.0003), and (MD = 0.06, 95% CI = 0.05–0.06, *p* < 0.00001), respectively. Also, the analysis showed a statistically significant association between vamorolone and increased TTRW velocity and increased Height percentile for age compared with the glucocorticoid group (MD = −0.14, 95% CI = −0.26 to −0.01, *p* = 0.03) and (MD = 17.82, 95% CI = 3.89–31.75, *p* = 0.01), respectively.

**Conclusion:**

Our study revealed a significant association between vamorolone and increased TTSTAND velocity, TTRW velocity, and TTCLIMB velocity compared with the placebo (steroid naïve), also showed a statistically significant association between increased TTRW velocity and increased Height percentile for age compared with the glucocorticoid that enhances the privilege of vamorolone over glucocorticoid in treating DMD patients. More multicenter randomized studies are needed to support our results.

## Introduction

Duchenne muscular dystrophy (DMD), an X-linked progressive neuromuscular condition, has reported a worldwide incidence of 1 in 3,500 to 1 in 5,000 live male births which equals 200 per million births, the onset appears in early childhood and ends with death in late teens (1). It is caused by mutations in DMD (encoding dystrophin) that prevent the production of the muscle isoform of dystrophin (Dp427m) (2). DMD is a severe, progressive muscle-wasting disease with early symptoms that includes difficulties in climbing stairs, a waddling gate, and repeated falls; patients first develop these symptoms between 2 and 3 years old. Most patients become wheelchair-dependent around the age of 10–12 years and require assisted ventilation at ~20 years of age. With optimal care, most DMD patients die between the age of 20 and 40 years from cardiac and/or respiratory failure (3).

Current medical treatment focuses on symptoms linked to the degeneration-regeneration cycle. Corticosteroids are considered the gold standard therapy for DMD and potentially effective in symptom reduction, while the exact mechanism is unknown, corticosteroids are supposed to predominantly reduce inflammation (4, 5). A systematic review revealed that prednisone enhances strength and pulmonary function in patients with DMD. Additionally, prednisone can improve motor function, postpone cardiomyopathy, and decrease the need for scoliosis surgery. Additional corticosteroids might also be beneficial in DMD; Deflazacort has the same effectiveness as prednisone with the distinct benefit of increasing survival at 5–15 years of follow-up (6–8). Unfortunately, glucocorticoid therapy is attributed to side effects like immunosuppression, muscle weakness, and Cushingoid look, as well as long-term harmful sequelae like osteopenia and stunted growth (9). Several corticosteroids, such as prednisone and deflazacort, act as agonists of the mineralocorticoid receptor, raising blood pressure, and volume *via* the renin-angiotensin system. In contrast, vamorolone is a strong antagonist of the mineralocorticoid receptor, similar to eplerenone and spironolactone in activity (9).

Vamorolone has a different mechanism of action from traditional corticosteroid anti-inflammatory medications as it holds the distinct NF-B inhibitory (anti-inflammatory) activities while losing the gene transcriptional activities connected to glucocorticoid response element binding and activation. It also has powerful antagonist activity for the mineralocorticoid receptor (5, 10–12). Vamorolone and corticosteroids both suppress the NF-B-related cell damage pathways, which are known to be one of the initial molecular disorders of dystrophin-deficient muscle in (DMD) patients (13). Previous studies (14–16) showed that vamorolone may be a safer alternative than prednisone as fewer physician-reported adverse effects (AEs) occurred with vamorolone than have been reported for treatment with prednisone and deflazacort, moreover that vamorolone therapy did not result in the growth stunting that is common with these corticosteroids indicating that vamorolone might be a suitable option for treating DMD. And to verify these findings, we performed a meta-analysis to determine whether vamorolone is more effective than corticosteroids.

## Methods

Preferred Reporting Items for Systematic Reviews and Meta-Analyses (PRISMA) guidelines and the Cochrane handbook were followed to perform this meta-analysis (17).

### Study design

This is a meta-analysis study that aimed to investigate the efficacy of vamorolone vs. placebo (steroid naïve) and corticosteroids for the treatment of DMD patients.

### Search strategy

(Vamorolone) AND (Glucocorticoids OR Prednisone OR placebo) AND (Duchenne muscular dystrophy OR Becker's Muscular Dystrophy OR Cardiomyopathy, Dilated, X-Linked OR Childhood Pseudo hypertrophic Muscular Dystrophy), we looked for relevant randomized control trials and observational studies in PubMed, Web of Science, Scopus and Cochrane library databases from inception to 17 November 2022.

### Eligibility criteria

Any randomized control trials and controlled observational studies such as Cross-sectional, prospective, or retrospective cohort and case-control studies that investigated the efficacy of vamorolone vs. placebo (steroid naïve) and corticosteroids for the treatment of DMD patients, so the PICO will be:

1- Population: DMD patient.
2- Intervention: vamorolone.
3- Control: placebo (steroid naïve) or corticosteroids.
4- Outcome: TTSTAND velocity, TTRW velocity, TTCLIMB velocity, Height percentile for age, and BMI Z score.

### Exclusion criteria

We exclude animal studies, case reports, case series, editorials, and reviews.

### Study selection process

Two independent authors (RHE and KRM) revised the titles and/or abstracts of the searched papers to determine suitable studies. Then, the two authors revised the full texts of the retrieved reports independently. Any conflicts between authors were solved by the first author.

### Data extraction and management

Two independent authors used an excel sheet to extract the following data: the first author's name, year of publication, age, sex, study design, country of the study, number of patients, Outcome measurements: TTSTAND velocity, TTRW velocity, TTCLIMB velocity, Height percentile for age and BMI Z score in Vamorolone vs. Placebo (steroid naïve) and Vamorolone vs. Glucocorticoids. Two authors performed data extraction of outcomes and any conflicts were solved by the first author (RHE).

### Quality assessment

Newcastle Ottawa scale tool was used to assess the quality because most of the included studies were non randomized studies; each study was given a score and ranked as good, fair, or poor quality.

### Data synthesis

Data were analyzed using RevMan software, version 5.4. Sensitivity analysis (leave-one-out test) was used. Continuous data were presented as mean difference (MD) with a 95% confidence interval (CI). If no heterogeneity was observed, results were presented in a fixed effect model and a random effect model was used if significant heterogeneity was observed. Results were considered significant if the *p* < 0.05.

## Results

### Summary of studies

**Figure 1:**
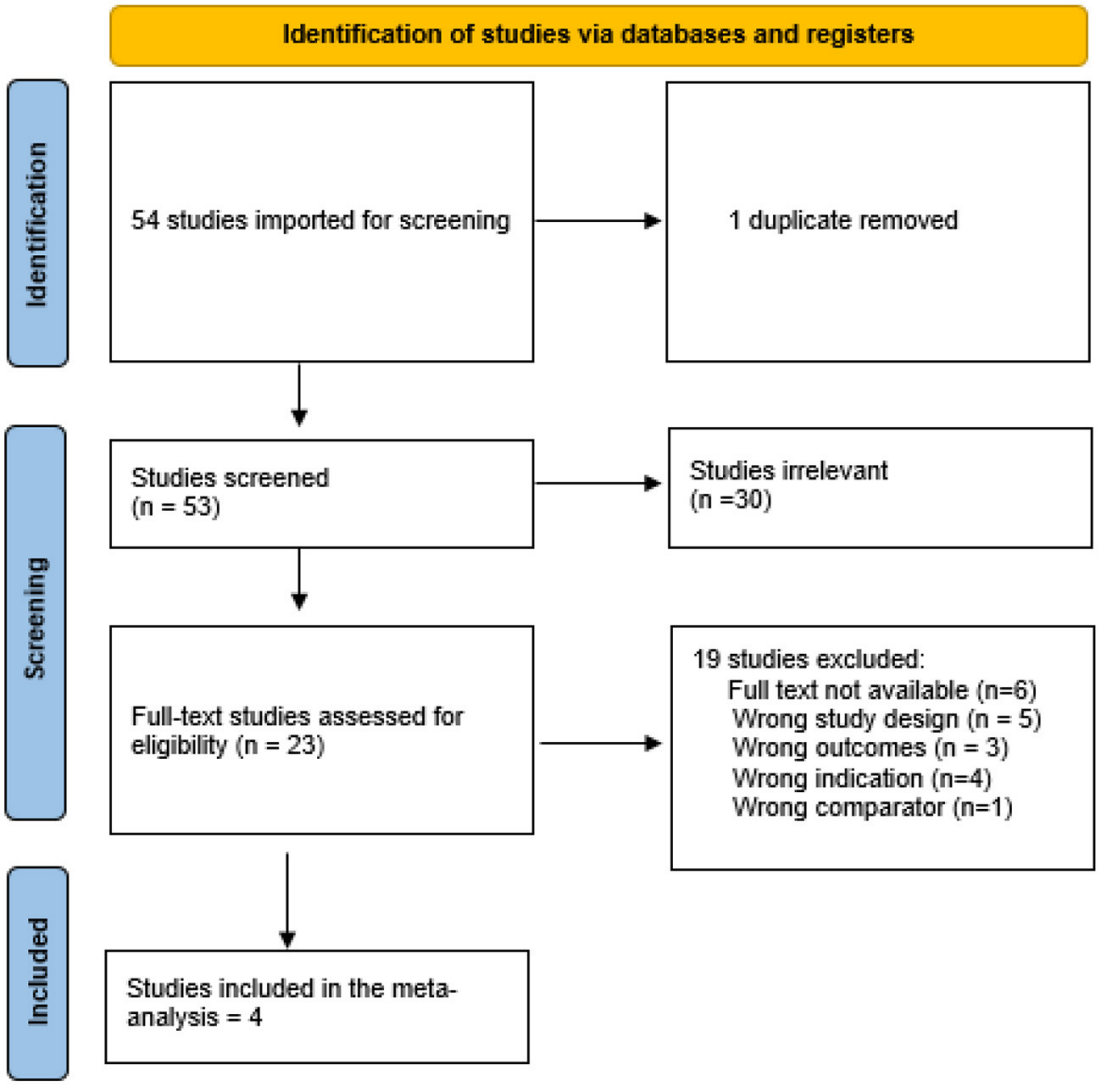
Prisma.

After a search of the literature, 54 studies resulted, and then became 53 were eligible for title and abstract screening after duplicate removal. Of the 53, 30 were irrelevant and 23 studies were eligible for full-text screening. Finally, four studies were included in the meta-analysis after the full-text screening, as shown in the PRISMA in Figure 1.

The overall quality was good in the four included studies, as shown in Table 1.

**Table 1:** Quality assessment.

	**Selection**				**Comparability**	**Exposure**			**Total point**	
**ID**	**(1) Represen** **tativeness**	**(2) Selection of the non-exposed cohort**	**(3) Ascertainment of exposure**	**(4) Demonstration that outcome of interest was not present at start of study**	**(1) Comparability of cohorts**	**(1) Assessment of outcome**	**(2) Was follow-up long enough for outcomes to occur**	**(3) Adequacy of follow up of cohorts**		**AHRQ Standards**
Smith et al. [16]	1	1	1	0	2	1	1	1	8	Good
Mah et al. [15]	1	1	1	0	1	1	1	1	7	Good
Hoffman et al. [29]	1	1	1	0	2	1	1	1	8	Good
Guglieri et al. [14]	1	1	1	0	2	1	1	1	8	Good

The total number of patients included in the study is 277 patients, 125 patients in the vamorolone group, 106 in the glucocorticoids group, and 46 in the placebo (steroid naïve) group. Other baseline data are shown in Table 2.

**Table 2:** Baseline characteristics.

		**Hoffman et al. (29)**	**Smith et al. (16)**	**Mah et al. (15)**	**Guglieri et al. (14)**
Number of patients in each group	Vamorolone	48	23	23	58
	GC	14	80	185	31
	Placebo or GC naivee	31	19	N/A	28
Age (years)	Vamorolone	4.9 ± 0.9	5.2 ± 0.90	5.83 ± 0.88	5.4 ± 0.9
	GC	5.7 ± 0.7	5.9 ± 0.65	6.03 ± 0.8	5.5 ± 0.9
	Placebo or GC naivee	4.9 ± 0.8	5.03 ± 0.55	N/A	5.4 ± 0.8
Weight in kg	Vamorolone	N/A	19.5 ± 2.5	21.98 ± 3.78	19 ± 3.5
	GC		20.53 ± 3.4	20.35 ± 3.55	21 ± 3
	Placebo or GC naivee		18.3 ± 2	N/A	20 ± 3
Height in cm	Vamorolone		107 ± 6.8	111.80 ± 6.94	26.6 ± 27.14
	GC		109.4 ± 5.8	109.86 ± 6.86	37 ± 29
	Placebo or GC naivee		105.4 ± 5.1	N/A	33 ± 29
BMI (kg/m^2^)	Vamorolone	16.9 ± 1.2	17 ± 0.9	17.68 ± 1.23	16.4 ± 1.3
	GC	16.3 ± 1.7	17.1 ± 1.9	16.68 ± 1.55	16.8 ± 1.3
	Placebo or GC naivee	15.2 ± 1.6	16.4 ± 0.9	N/A	16.8 ± 1.1
TTSTAND	Vamorolone	M = 0.0452 (SD = 0.053)	M = 0.241 (SD = 0.076)	M = 0.2 (SD = 0.13)	M = 0.0448 (SD = 0.0804)
	GC	M = 0.01 (SD = 0.068)		M = 0.25 (SD = 0.13)	
	Placebo or GC naivee		M = 0.205 (SD = 0.102)		M = −0.01 (SD = 0.06)
TTRW	Vamorolone	M = 0.1604 (SD = 0.2508)	M = 2.061 (SD = 0.347)	M = 1.87 (SD = 0.63)	M = 0.2212 (SD = 0.2612)
	GC	M = −0.01 (SD = 0.256)		M = 1.89 (SD = 0.71)	
	Placebo or GC naivee		M = 1.717 (SD = 0.46)		M = 0.02 (SD = 0.33)
TTCLIMB	Vamorolone	M = 0.0448 (SD = 0.0759)	M = 0.331 (SD = 0.127)	M = 0.32 (SD = 0.19)	M = 0.0619 (SD = 0.0198)
	GC	M = 0.01 (SD = 0.062)		M = 0.33 (SD = 0.18)	
	Placebo or GC naivee		M = 0.242 (SD = 0.108)		M = 0.0056 (SD = −0.0078)
Height percentile for age	Vamorolone		M = 35.24 (SD = 29.82)	M = 37.03 (SD = 31.14)	M = 2.026 (SD = 8.003)
	GC		M = 26.14 (SD = 24.21)	M = 13.42 (SD = 18.62)	M = −1.88 (SD = 8.81)
	Placebo or GC naivee		M = 27.16 (SD = 21.17)		
BMI *z* score	Vamorolone	M = 0.2582 (SD = 0.5576)	M = 1.46 (SD = 0.62)	M = 1.52 (SD = 0.66)	M = 0.46 (SD = 0.54)
	GC	M = 0.543 (SD = 0.6646)	M = 1.068 (SD = 1.05)	M = 0.79 (SD = 1.11)	M = 0.41 (SD = 0.51)
	Placebo or GC naivee		M = 0.36 (SD = 0.77)		

### Outcomes

#### Vamorolone vs. placebo

##### TTSTAND velocity

**Figure 2:**
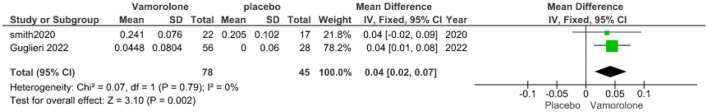
TTSTAND outcome vamorolone vs. placebo.

The pooled analysis showed a statistically significant association between the vamorolone group and increased TTSTAND velocity compared with the placebo group (MD = 0.04, 95% CI = 0.02–0.07, *p* = 0.002). We observed no significant heterogeneity between the two studies (*P* = 0.79, *I*^2^ = 0%, Figure 2).

##### TTRW velocity

**Figure 3:**
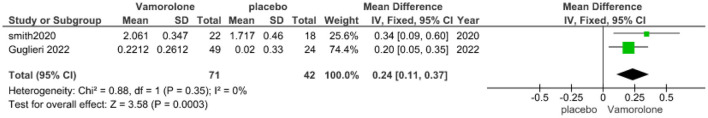
TTRW outcome vamorolone vs. placebo.

The pooled analysis showed a statistically significant association between the vamorolone group and increased TTRW velocity compared with the placebo group (MD = 0.24, 95% CI = 0.11–0.37, *p* = 0.0003). We observed no significant heterogeneity between the two studies (*P* = 0.35, *I*^2^ = 0%, Figure 3).

##### TTCLIMB velocity

**Figure 4:**
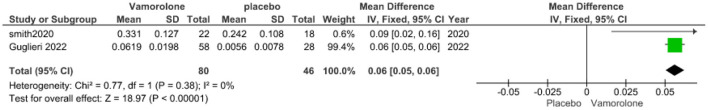
TTCLIMB vamorolone vs. placebo.

The pooled analysis showed a statistically significant association between the vamorolone group and increased TTCLIMB velocity compared with the placebo group (MD = 0.06, 95% CI = 0.05–0.06, *p* < 0.00001). We observed no significant heterogeneity between the two studies (*P* = 0.38, *I*^2^ = 0%, Figure 4).

#### Vamorolone vs. glucocorticoids

##### TTSTAND velocity

**Figure 5:**
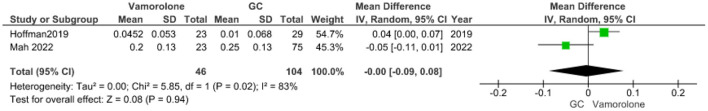
TTSTAND outcome vamorolone vs. glucocorticoids.

The pooled analysis showed no statistically significant difference between the vamorolone group and the Glucocorticoid group (MD = 0.00, 95% CI = −0.08–0.09, *p* = 0.94). We observed a significant heterogeneity between the two studies (*P* = 0.02, *I*^2^ = 83%, Figure 5).

##### TTRW velocity

**Figure 6:**
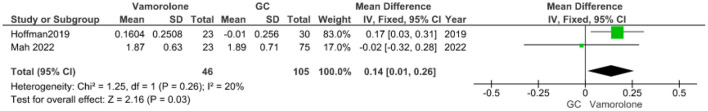
TTRW outcome vamorolone vs. glucocorticoids.

The pooled analysis showed a statistically significant association between the vamorolone group and increased TTRW velocity compared with the glucocorticoid group (MD = −0.14, 95% CI = −0.26 to −0.01, *p* = 0.03). We observed no significant heterogeneity between the two studies (*P* = 0.26, *I*^2^ = 20%, Figure 6).

##### TTCLIMB velocity

**Figure 7:**
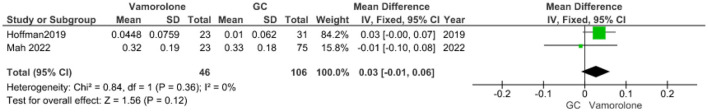
TTCLIMB outcome vamorolone vs. glucocorticoids.

The pooled analysis showed no statistically significant difference between the vamorolone group and the glucocorticoid group (MD = −0.03, 95% CI = −0.06–0.01, *p* = 0.12). We observed no significant heterogeneity between the two studies (*P* = 0.36, *I*^2^ = 0%, Figure 7).

### Height percentile for age

**Figure 8:**
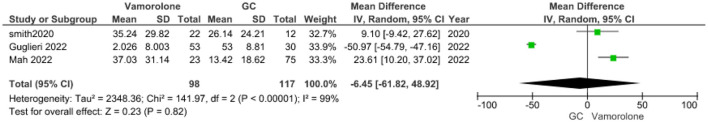
Height percentile for age outcome vamorolone vs. glucocorticoids.

The pooled analysis showed no statistically significant difference between the vamorolone group and the glucocorticoid group (MD = −6.45, 95% CI = −61.82–48.92, *p* = 0.82). We observed a significant heterogeneity among the studies (*P* < 0.00001, *I*^2^ = 99%). It was solved by leave-one-out test by removing ((14); *P* = 0.21, *I*^2^ = 35%), and the analysis showed a statistically significant association between the vamorolone group and increased height percentile for age compared with the glucocorticoid group (MD = 17.82, 95% CI = 3.89–31.75, *p* = 0.01, Figure 8).

### BMI *Z* score

**Figure 9:**
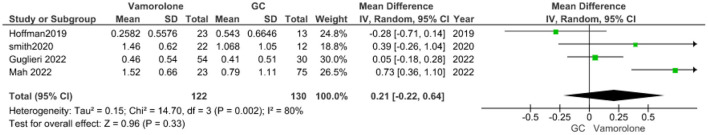
BMI z score outcome vamorolone vs. glucocorticoids.

The pooled analysis showed no statistically significant difference between the vamorolone group and the glucocorticoids group (MD = 0.21, 95% CI = −0.22–0.64, *p* = 0.33). We observed a significant heterogeneity among studies (*P* = 0.002, *I*^2^ = 80%). It was solved by leave-one-out test by removing ((15); *P* = 0.19, *I*^2^ = 39%), and the analysis showed no statistically significant difference between the vamorolone group and glucocorticoid group (MD = −0.01, 95% CI = −0.29–0.30, *p* = 0.97, Figure 9).

## Discussion

The pooled analysis revealed a statistically significant association between the vamorolone group and increased TTSTAND velocity, increased TTRW velocity, and increased TTCLIMB velocity compared with the placebo group. Similarly, the pooled analysis showed a statistically significant association between the vamorolone group and increased TTRW velocity compared with the glucocorticoid group. However, the pooled analysis showed no statistically significant difference between the vamorolone group and the glucocorticoid group regarding TTSTAND velocity and TTCLIMB velocity. Also, the analysis showed a statistically significant association between the vamorolone group and increased height percentile for age compared with the glucocorticoid group. Additionally, the pooled analysis showed no statistically significant difference between the vamorolone group and glucocorticoid group regarding BMIZ score.

One of the initial molecular pathologies of dystrophin-deficient muscle in Duchenne muscular dystrophy (DMD) patients is known to be the activation of NFκB-related cell damage pathways, and vamorolone and corticosteroids both suppress these pathways (13). Numerous inflammatory genes are regulated by NFκB in immune cells as well as muscle fibers (18–21) and the invasion and activation of these cells can result in the death of muscle cells (22, 23). Vamorolone is a first-in-class steroidal anti-inflammatory drug (24), it lacks an 11-carbon oxygen group (hydroxyl or carbonyl), one of five molecular interaction sites with the glucocorticoid receptor, which sets it apart from the other 33 medications in the corticosteroid class (25, 26). Vamorolone retains the anti-inflammatory properties of steroid medications while lacking its side effects (AEs), such as growth retardation, bone morbidities, and muscular atrophy (25, 27). Vamorolone inhibiting NFκB-associated proinflammatory signals in a ligand/receptor monomeric state rather than the more conventional molecular models of ligand/receptor dimeric complexes is consistent with the retention of anti-inflammatory activity and absence of side effects in preclinical models (28). Numerous corticosteroids, such as prednisone and deflazacort, act as agonists of the mineralocorticoid receptor, raising blood pressure and volume *via* the renin-angiotensin system. In contrast, vamorolone has the same activity as eplerenone and spironolactone as a powerful antagonist of the mineralocorticoid receptor (11). Vamorolone optimizes traditional steroidal anti-inflammatory activities: retains the distinct NFκB inhibitory (anti-inflammatory) activities while losing the gene transcriptional activities connected to glucocorticoid response element binding and activation, is a powerful antagonist for the mineralocorticoid receptor, and has superior membrane stabilization properties (5, 10–12).

Our findings agree with the findings of Hoffman et al. (29) who concluded that oral administration of vamorolone in individuals with DMD over the course of a 24-week therapy term was safe, effective, and well-tolerated at all tested doses. When they used vamorolone at doses of 2.0 and 6.0 mg/kg/d for a period of 24 weeks, the drug met the primary efficacy outcome of improved muscle function. They also discovered that the majority of the negative effects of glucocorticoids are not present in vamorolone including bone turnover and insulin resistance improvement and less weight gain. Serum osteocalcin is a reliable indicator of bone production and turnover in children that is essential to growth (30). Improvements in bone density and bone geometry have been demonstrated to be predicted by increases in serum osteocalcin (31). Prednisone and deflazacort both dramatically lower serum osteocalcin levels (32, 33). Vamorolone, on the other hand, boosted osteocalcin levels, indicating a reduction of deleterious bone effects (29). The lack of deleterious alterations in bone turnover indicators in DMD patients receiving vamorolone treatment raises the possibility of losing the bone morbidities usually associated with glucocorticoids (29). They also found evidence of adrenal suppression (29).

Our results also support those of Guglieri et al. (14), who discovered that vamorolone is effective and safe in the treatment of boys with DMD at a wide dose range (2–6 mg/kg per day) and throughout a 24-week course of therapy. They also discovered that vamorolone is a safer alternative than prednisone in this disease as it is a dissociative steroid, which means that it isolates safety issues (growth deceleration, bone biomarkers abnormalities) from efficacy (improving motor results in DMD). Their results confirmed earlier research that showed vamorolone-treated DMD youngsters had normal development trajectories across 18 months (16) and 30 months (15). Prednisone medication, on the other hand, reduced development trajectories in this 24-week experiment, supporting numerous studies of corticosteroid treatment in DMD (15, 34, 35). Furthermore, neither of the vamorolone dose groups showed mean reductions in any of the bone turnover markers, supporting the enhanced safety profile of vamorolone on bone health. The favorable bone biomarker profile observed in the vamorolone-treated groups compared with corticosteroids is explained by vamorolone not being a substrate for 11β-hydroxysteroid dehydrogenase enzymes which are necessary for corticosteroid-induced bone morbidities in mice, as it lacks the 11βmoiety acted upon by these enzymes (36, 37). Additionally, Guglieri et al. incidentally found an unexpectedly high incidence of adrenal insufficiency at baseline in boys with DMD in both vamorolone-treated and corticosteroids-treated groups using both ACTH-stimulation and morning cortisol measurements, as corticosteroid medications (including endogenous cortisol) potently, widely, and rapidly block the hypothalamic-pituitary-adrenal (HPA) axis and long-term use can result in adrenal insufficiency (38), this finding with the vamorolone-treated group requires additional investigation.

Similar to our findings, Mah et al. (15) discovered that among boys with DMD who were 4–7 years old at enrolment, vamorolone therapy was not connected to a change in TTSTAND velocity from baseline to 30 months. However, long-term vamorolone therapy at doses up to 6.0 mg/kg/d showed to be safe and effective based on clinical and laboratory outcomes. Additionally, over a 30 month treatment period, subjects receiving larger doses of vamorolone (i.e., ≥2 mg/kg/d) continued to show improvement in their motor function as measured by the TTCLIMB, TTRW, NSAA, and 6MWT distance. They also discovered that boys treated with vamorolone experienced significantly less bone age delay in relation to chronological age than boys treated with corticosteroid therapy. Additionally, unlike traditional corticosteroid medication, vamorolone does not have the same association with insulin resistance; however, long-term vamorolone therapy may be linked to adrenal suppression (16). Similarly, Smith et al. (16) discovered that over the course of the 18 month treatment period, boys receiving vamorolone showed improvements from baseline in all five motor evaluations (TTSTAND, TTRW, TTCLIMB, NSAA, and 6MWT). Contrary to those who received corticosteroids, those who received vamorolone did not show any signs of growth stunting. Moreover, comparing published trials of deflazacort- and prednisone-treated DMD patients, doctors found that participants receiving vamorolone experienced fewer other corticosteroid-related safety issues, such as weight gain, Cushingoid appearance, behavior change (mood disturbance), and hirsutism.

## Future implications

Our analysis results revealed that vamorolone treatment was associated with improvements in some motor outcomes like TTRW velocity and increased height percentile for age compared with the glucocorticoid group. Our analysis suggests that vamorolone can be used in the treatment of patients with DMD as it is more effective and safer than corticosteroids.

## Strengths and limitations

The overall quality was good in all of the studies included in our analysis. The short study period and small sample size are limitations of our analysis. In addition, most of our studies were clinical trials but not randomized. Therefore, to support our findings and further evaluate the efficacy and safety of vamorolone treatment in DMD patients, prospective randomized clinical trial studies with larger sample sizes and longer follow-up durations are required.

## Conclusion

Our pooled analysis revealed a statistically significant association between the vamorolone group and increased TTSTAND velocity, increased TTRW velocity, and increased TTCLIMB velocity compared with the placebo group. Similarly, the pooled analysis showed a statistically significant association between the vamorolone group and increased TTRW velocity, and increased height percentile for age compared with the glucocorticoid group. However, the pooled analysis showed no statistically significant difference between the vamorolone group and the glucocorticoid group regarding TTSTAND velocity, TTCLIMB velocity, and BMIZ score. Therefore, we suggest that vamorolone can be used in treating patients with DMD. More randomized clinical trials are needed to support our findings.

## Data availability statement

The original contributions presented in the study are included in the article/supplementary material, further inquiries can be directed to the corresponding author.

## Author contributions

RE: idea-screening-data extraction and analysis. KM: screening-quality assessment and writing. NT: data extraction and writing. SR: writing. JS: screening and reviewing. All authors contributed to the article and approved the submitted version.
